# Internet Exposure Associated With Canadian Parents’ Perception of Risk on Childhood Immunization: Cross-Sectional Study

**DOI:** 10.2196/publichealth.8921

**Published:** 2018-01-19

**Authors:** Jordan Lee Tustin, Natasha Sarah Crowcroft, Dionne Gesink, Ian Johnson, Jennifer Keelan

**Affiliations:** ^1^ School of Occupational and Public Health Ryerson University Toronto, ON Canada; ^2^ Dalla Lana School of Public Health University of Toronto Toronto, ON Canada; ^3^ Public Health Ontario Toronto, ON Canada; ^4^ Laboratory Medicine and Pathobiology University of Toronto Toronto, ON Canada; ^5^ Concordia University of Edmonton Edmonton, AB Canada

**Keywords:** Canadian parents, vaccination, immunization, Internet, vaccine safety

## Abstract

**Background:**

There is a large presence of provaccination and antivaccination content on the Internet. The Internet has been identified as an important source for parents to seek and share vaccine information. There are concerns that parental fears or hesitancy on childhood immunizations are increasing due to the popularity of social media and exposure to online antivaccination sentiment. No other studies have investigated the association between seeking vaccine information online and Canadian parents’ perception of risk on childhood immunization.

**Objective:**

We aimed to investigate the potential association between seeking vaccine information on the Internet and Canadian parents’ perception of risk on childhood immunization in order to quantify the perceived association and increase our understanding on the impact of the Internet to help guide public health interventions.

**Methods:**

We analyzed this association in two population samples: a self-selecting Web-based sample of Canadian parents recruited through Facebook (n=966) and a population-based sample of parents recruited by random digit dialing (RDD; n=951). The outcome was parental perception of vaccine safety on a seven-point ordinal scale from “not safe” to “extremely safe.” An ordinal regression model was used to investigate if Internet information seeking on childhood vaccination predicted parental perception of vaccine safety.

**Results:**

After adjusting for income level, Internet reliability, age of parent, and region, the odds of perceiving vaccines as less safe rather than more safe were 1.6 times higher (95% CI 1.3-2.1) for parents who used the Internet to search for vaccination information compared to parents who did not search the Internet in the Web-based sample, and 2.0 times higher (95% CI 1.6-2.5) in the population-based RDD sample.

**Conclusions:**

The results suggest the Internet is significantly associated with Canadian parents’ negative perception of vaccine risk. Governmental and scientific sectors should consider the development and implementation of Web-based vaccine interventions to promote confidence in immunization.

## Introduction

A decrease in public confidence in the safety of vaccines and subsequent lower vaccine uptake has been described as an “impending crisis” in the developed world [1,2]. In Canada, the public’s confusion and doubt over the measles-mumps-rubella (MMR) vaccine was highlighted by a 2010 study reporting that 65% of women and 72% of men believe the vaccine is unsafe or are unsure whether or not the vaccine could cause autism [3]. In addition, a 2015 survey revealed that two in five Canadians believe “the science on vaccinations isn’t quite clear” [4]. In 2011, a national survey of Canadians revealed suboptimal coverage rates for childhood immunizations [5], and several measles outbreaks have been reported across Canada since 2011 [6,7]. In 2014 and 2015, the Public Health Agency of Canada (PHAC) released a public health notice warning Canadians of the unusually high number of measles cases in Canadian provinces [8-10]. The most recent report on measles trends in Canada found that areas of low immunization coverage and case importations are presenting a challenge to Canada’s measles elimination status [11]. In 2017, several outbreaks of mumps were reported across Canada, prompting the Chief Public Health Officer to issue a statement reminding Canadians on the importance of vaccination [12].

The popularity of social media has been identified by the public health community as one of the reasons for the increase in parental fears about childhood vaccines because the Internet is an important vehicle for individuals seeking health information and support, and sharing health knowledge, opinions, and experiences [13]. For example, Statistics Canada reports that 80% of Canadians 16 years of age or older use the Internet [14]; 64% of these Internet users search for medical- or health-related information, with the majority of these Internet users between the ages of 16 to 44 years [15]. At the time of this study, Facebook is reported as the most popular social media platform in Canada. More than half of the population logs into Facebook at least once per month and Canadian usage rates are higher than global and US averages [16,17]. With the increasing popularity of social media, the public appears to be bypassing conventional sources of health information and looking for the “wisdom of the crowd,” where health decisions depend on other Internet users’ experiences [18]. The Internet allows for rapid sharing of opinions and information, self-organization, the creation of social networks, and empowerment of online groups or people such as antivaccine communities or activists [2]. The large presence of online antivaccination sentiment together with the current pattern of mistrust in the medical community has led to an environment of parents seeking and sharing immunization information [19]. A recent study investigating parents’ confidence in childhood vaccines in the United States found that both vaccine-declining and vaccine-accepting parents have questions, concerns, or misperceptions about vaccines [20]. The majority of parents reported seeking information about vaccine safety prior to vaccinating their children, and identified the Internet as an important source of information. The authors reported a need for the public health community to have a more informed understanding of parents’ Internet use, and of how and to what extent social media interactions with recognized public health organizations can address parents’ vaccine questions. Given the increasing popularity of social media platforms in Canada among Generation X and millennial parents, as well as the suggested influence of social media on parental beliefs and behaviors toward childhood immunization, it is important to investigate and understand this influence in order to inform Web-based interventions that could influence hesitant or undecided parents.

The Health Belief Model is widely applied to determine what factors influence individuals when making vaccination decisions. In terms of immunization, the decision to vaccinate is balanced by the perceived risk of contracting a vaccine-preventable disease and the perceived risk of vaccine adverse events. 

**Figure 1:**
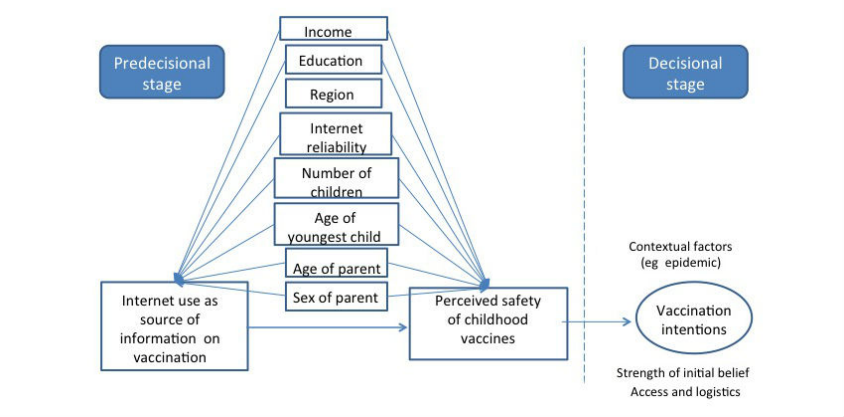
Conceptual model on the association between using the Internet to search for information on vaccinations and parental perception on safety of vaccinations.

Due to the abundance and availability of antivaccination sentiment online and the relatively low prevalence of vaccine-preventable disease in the population, it is suggested that individuals may perceive a greater risk of suffering from vaccination side effects than from contracting a vaccine-preventable disease [13]. Therefore, information obtained online that clarifies one’s understanding of vaccination risks should also affect the intent to vaccinate (Figure 1). This study investigates the impact of reported online vaccine information-seeking behaviors on perceived immunization risk in two different samples of Canadian parents. We hypothesized that parents who report seeking vaccination information online will perceive vaccines as less safe compared to parents who do not seek information online. Examining this association will increase our understanding and provide evidence on the impact of the Internet on parental perception of risk in the context of childhood immunization to help guide public health interventions.

## Methods

### Data Sources and Collection

We examined the potential association between seeking vaccine information on the Internet and Canadian parents’ perception of risk on childhood immunization data on two different data sources: primary data collected via Web-based survey and secondary data collected via population-based random digit dialing (RDD). We used two independent data sources with the same variables to test the association in two Canadian parent populations recruited at different times and via different methods. Both the Web-based and RDD survey contained questions on respondent demographics and knowledge, awareness, attitudes, and behaviors related to immunization. Identical questions to the RDD survey were used to measure the exposure, main outcome, and confounders in the primary data collection via Web-based survey, with the question format slightly altered for Web-based delivery.

We collected the Web-based survey data via targeted advertisement recruitment on Canada’s most popular social media platform, Facebook. French and English advertisements invited Canadian parents to click on the advertisement and participate in a Web-based survey on childhood immunization with a chance to win an iPad mini. Based on sample size calculations to detect an odds ratio of 1.5 and available budget, we aimed to recruit 800 participants. An odds ratio of 1.5 was used for two reasons: (1) to ensure sufficient sample size should the exposure be mildly but statistically associated with the outcome [21], and (2) the value of 1.5 was determined to be a meaningful increase from a public health standpoint and reasonable from an operational research standpoint. We piloted the survey with a convenience sample of 20 Facebook users and their “friends” before advertising to the larger Facebook population. For 4 weeks in December 2013 and January 2014, we displayed the advertisements on the newsfeed of users who were (1) located in Canada, (2) 18 years or older, and (3) parents of a child aged 0 to 15 years. Users who clicked on the advertisement were redirected to a secure Web-based survey, which contained details on the study, eligibility criteria, and informed consent. The survey automatically terminated if the respondent did not provide informed consent or did not meet eligibility criteria. We were successful in recruiting our targeted population via this method, as also reported by several recent studies that were successful using Facebook as a viable and cost-effective recruitment tool for health research and/or to reach targeted populations [22-27]. The survey response rate was 22.89% (1097 respondents/4792 unique Facebook users who clicked on the Facebook advertisements) and the survey completion rate was 64.68% (1097 respondents/1696 unique Facebook users who started the Web-based survey) with little missing data, resulting in a sample size of 1097 Canadian parents [28]. Further details on the methods and results of the recruitment strategy are available [28].

The population-based RDD data are secondary data deidentified and extracted from a survey collected by a reputable research company, EKOS Research Associates, contracted by PHAC. Experts in immunization and epidemiology at PHAC worked with the research company in the development and testing of the questionnaire. The objective was to collect descriptive data on Canadian parents’ knowledge, awareness, attitudes, and behaviors related to immunization to inform policy makers. The secondary data were collected via telephone survey on a population-based RDD sample of Canadian parents during a period of 3 weeks in March 2011. Respondent inclusion criteria were (1) 18 years of age or older, (2) parents of at least one child younger than 18 years, (3) resident of Canada, and (4) able to respond to questions in English or French. The research company compiled a summary report available online [29] and PHAC provided the raw data for the purposes of this study.

Researchers calculated the response rate based on the empirical method (completed + ineligible) / (unresolved + ineligible + nonresponding eligible + completed + nonresponding unknown) and reported a rate of 23·43% (7898/33,698) resulting in a sample size of 1745 Canadian parents [29]. Power calculations estimated 90% power to detect an odds ratio of 1.5 with 95% two-sided significance level.

Ethical approval was obtained from the University of Toronto’s Office of Research Ethics (REF#29309).

#### Primary Exposure

We classified respondents who sought out information on childhood vaccines and reported the Internet as one of their top three sources used for information on vaccines as “used the Internet” and those who do not seek out information on childhood vaccines or do not report the Internet as one of their top three sources as “did not use the Internet.”

#### Outcome

We measured respondent perception on vaccine safety as an ordinal variable from 1 to 7: 1=not at all safe, 4=moderately safe, and 7=extremely safe.

#### Potential Confounders

We hypothesized parental education level and income, parental age and sex, age of youngest child, number of children, place or residence, and the relative importance of the Internet as a source of information relative to the importance of family, friends, and/or a health care professional as potential confounders. We measured education level according to four levels: high school or less, trade or vocational school, some university, and bachelors/graduate degree/professional certification. We measured household income level in Can $10,000 increments ranging from less than Can $30,000 to Can $120,000 and we categorized the variable into four levels (less than Can $30,000, Can $30,000-$59,999, Can $60,000-$99,000, and more than Can $100,000) in order for sufficient sample size in each category and to make comparisons among intermediary groups from lowest to highest income. We measured parental age as continuous (years) in the Web-based survey and it was measured as a categorical variable in the RDD survey (younger than 30 years, 30-34 years, 35-39 years, 40-44 years, and 45 years and older). The age of youngest child was measured as continuous (years) in both surveys, and the number of children was measured as categorical (1, 2, 3, 4, 5, and 6 or more) in the Web-based survey and as continuous in the RDD survey. We classified the perceived reliability of the Internet relative to family, friends, or health care professionals as (1) “reported as most reliable and trustworthy source on vaccines” to (4) “not reported in respondent’s top three choices as a reliable source of information on vaccines.” We categorized place of residence into six regions due to low numbers and to reflect the regions reported in the RDD data: British Columbia, Alberta, Saskatchewan or Manitoba, Ontario, Québec, and Atlantic provinces or Territories.

### Statistical Analysis

We excluded participants with missing data from the analyses as sufficient power remained and differences were not detected on the primary independent and dependent variables [21]. We conducted descriptive statistics to describe the characteristics of both samples. We then conducted bivariate ordinal logistic regression to assess associations between each variable and the outcome, respondent perception of vaccine safety. We chose the largest category size as the reference category for categorical variables [21]. We used multivariate ordinal logistic regression modeling to assess the association between Internet use and respondent perception of vaccine safety. The ordinal regression modeled the cumulative odds of perceiving vaccines as “not safe” using the seven-point ordinal outcome variable. As proposed by Hosmer and Lemeshow [30], we used the purposeful selection algorithm to select covariates to retain in the final predictive models. The method uses purposeful variable entry and retention parameters that retain significant covariates but also important confounding variables [30,31]. We included all variables significant at *P* ≤.25 in the multivariable analyses because more traditional levels (eg, .05) can miss important confounding variables [32]. We tested interaction terms of all possible two-way interaction terms against a reduced model using the likelihood ratio test and, in the first analysis, we considered all interaction terms for removal from the model as a block and contrasted against the model with all the main effects but without interaction terms [33]. We removed covariates from the multivariable model if they were not statistically significant at the .1 alpha level and not a confounder. We measured confounding as a 15% or greater change in the parameter estimate of our main association in the reduced model compared to the full model [30]. We utilized purposeful entry and retention parameters, including the choice of the 15% change-in-parameter-estimate criterion, due to the lack of prior information on known confounders for the investigated association [34]. At the end of this iterative process, we added any variable not entered into the original full model back in one at a time to further assess confounding [30]. This step can help to identify confounders that may not have been significant independently, yet make an important contribution in the presence of other variables [31]. We performed model diagnostics to rule out multicollinearity among covariates, to test for departure from linearity, and to examine the effect of influential observations and variables on our final models. The score test for the proportional odds assumption can be over conservative with large sample sizes or in multivariable analyses, thus we tested the proportional odds assumption by comparing the cumulative odds ratios in a series of six binary logistic models [35]. The assumption held as the odds ratios were all in the same direction and of approximately similar magnitude [35]. We decided to further validate the models from ordinal regression by also conducting binary logistic regression by categorizing the seven-point ordinal variable into a dichotomized outcome variable (levels 1-4: not safe to moderately safe; levels 5-7: safe to extremely safe) We utilized those cut-offs because levels 1 to 4 could be indicative of vaccine hesitancy and concerns with vaccination, whereas levels 5 to 7 indicated confidence in vaccines. We assessed model fit with Pearson and deviance goodness-of-fit statistics (and the Hosmer-Lemeshow test for the binary models) [21]. Although multivariable analyses using non-weighted data produced similar results, we utilized complex sampling procedures available in SAS version 9.3 for descriptive and multivariable analyses of the RDD data to reflect the complex survey design and population weights. We conducted all data analyses using SAS version 9.3 (SAS Institute Inc, Cary, NC, USA).

## Results

### Descriptive Statistics

Both samples had similar education and income level distributions with almost half of the respondents following the education distribution of Canadian adults by completing some level of higher education [36], and the majority being close to or above the 2012 median total household income of Can $74,540 for Canadian families [37]. In the Web-based sample, approximately half of respondents reported higher education with a university degree or professional certification, and 38.5% (379/985) reported an income greater than Can $100,000, followed by 35.6% (351/985) reporting an income of Can $60,000 to Can $99,999. In the population-based RDD sample, 42.19% (722/1738) of respondents reported a bachelor’s degree or higher, and 33.50% (519/1559) reported an income greater than Can $100,000, followed by 32.09% (498/1559) reporting an income of Can $60,000 to Can $99,999. The distribution on place of residency was similar in both samples; however, the Web-based sample had a lower proportion of Québec residents (10.96%, 120/1097 vs 24.26%, 427/1745) and a higher proportion of Alberta residents (23.65%, 259/1097 vs 10.17%, 200/1745). In both samples, approximately one-third of the respondents were Ontario residents, which corresponds to the Canadian geographic distribution as it is estimated that 38.5% of Canadians reside in Ontario [38]. There were noted differences in the distributions of parental age and sex, and age of youngest child in the two samples. In the Web-based sample, the mean age of respondents was 32 (SE 3.78) years and the median age of their youngest child was 2 (IQR 1.0) years.

The majority of Web-based respondents (68.77%, 751/1092) were younger than 35 years, female (92.61%, 1003/1083), and reported two or fewer children (81.49%, 894/1097). In the population-based RDD survey, the majority of respondents (62.29%, 674/1082) were 40 years or older and the mean age of their youngest child was 8.3 (SE 0.1) years. In addition, 41.02% (711/1745) were male and the median number of children per respondent was 2 (IQR 1.0).

**Figure 2:**
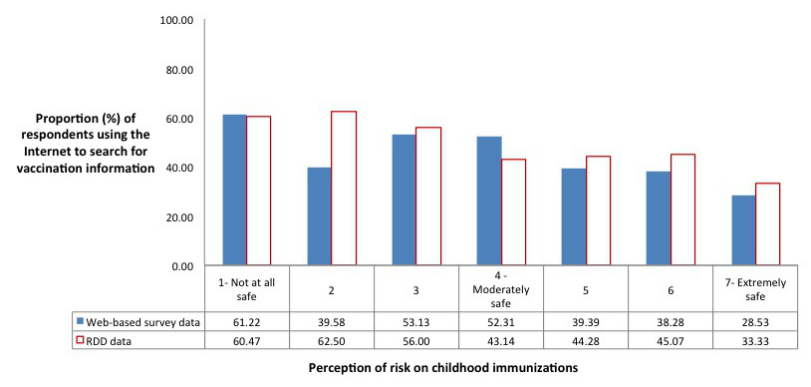
Perception of risk of childhood immunizations in parents who used the Internet to search for information on immunizations (Web-based survey data: n=1086; RDD data: n=1713).

For both data sources, approximately one-quarter of the respondents reported the Internet to be a reliable source for information on vaccines or vaccination, and approximately 40% (39.10%, 427/1092 vs 41.57%, 716/1729) reported using the Internet to search for information on vaccines. In terms of perception on safety of childhood immunizations, 26.77% (292/1091) of the Web-based survey respondents and 18.74% (324/1729) of the RDD survey respondents reported childhood immunizations as not at all safe to moderately safe (Table 1). A significant linear trend (Cochrane-Armitage tests for trend *P*<.001) was found between looking for information on the Internet and perception of risk of childhood immunizations for both data sources. Note that 11 respondents in the Web-based survey data and 32 respondents in the RDD data were excluded due to missing data (Figure 2).

### Multivariable Analysis

#### Web-Based Survey Data

Complete data were available for 966 respondents. The variables sex of parent and age of youngest child were removed from the multivariable analysis due to nonsignificance in bivariate analyses. Multicollinearity was not present and no interaction terms were retained due to nonsignificance of the likelihood ratio test between the model with all possible covariates and two-way interaction terms and the reduced model without interaction terms. Thus, ordinal logistic regression was performed with the following full model: Internet use, education level, income level, age of parent, age of youngest child, region, and reliability of the Internet. Nonsignificant variables (education level, number of children, and income level) were tested for potential confounding with only income level being retained in the model due to a significant change (26%) in the predictor’s estimate compared to the full model excluding education level and number of children. Originally excluded variables (sex of parent and age of youngest child) were individually re-entered into the model and were not found to be significant confounders. The covariates income level, Internet reliability, age of parent, and regions of residence remained in the final model (Table 2). After adjusting for income level, Internet reliability, age of parent, and region, the odds of perceiving vaccines as less safe rather than safe are 1.6 times higher (95% CI 1.3-2.1) for parents who use the Internet to search for vaccination information compared to parents who do not search the Internet. Chi-square statistics (deviance *P*>.99, Pearson *P*=.10) indicated model fit. Furthermore, the binary logistic regression produced similar estimates and precision (OR 1.6, 95% CI 1.1-2.3), and good model fit (Hosmer and Lemeshow *P*=.09).

#### Population-Based Random Digit Dialing Data

Complete data were available for 951 RDD respondents. The variables sex of parent and income level were removed from the multivariable analysis due to nonsignificance in bivariate analyses. Multicollinearity was not present and all interactions terms were removed from the model. No interaction terms were retained due to nonsignificance of the likelihood ratio test between the model with all possible covariates and two-way interaction terms and the reduced model without interaction terms. Thus, ordinal logistic regression was performed with the following full model: education level, age group of parent, age of youngest child, number of kids, region, and reliability of the Internet. Nonsignificant variables in the full model (education level, number of children, age of youngest child, and age group of parent) were tested for potential confounding with only age group of parent being retained in the model due to a significant change (21.7%) in the predictor’s estimate compared to the full model excluding education level, number of children, and age of youngest child. All originally excluded variables (sex of parent and income level) were individually re-entered into the reduced model to check for confounding, and income level was then retained in the final model due to a significant change (16%) of the predictor’s estimate (Table 2). After adjusting for income level, Internet reliability, age of parent, and region, the odds of perceiving vaccines as less safe rather than safe are 2.0 times higher (95% CI 1.6-2.5) for parents who use the Internet to search for vaccination information compared to parents who do not search the Internet. Chi-square statistics (deviance *P>*.99, Pearson *P*>1.0) indicated model fit. Binary logistic regression produced similar estimates and precision (OR 2.2, 95% CI 1.5-3.1), and good model fit (Hosmer and Lemeshow *P*=.63).

**Table 1:** Characteristics of both study samples for continuous and categorical variables.

Characteristic		Web-based survey (n=1097)	Population-based RDD survey (n=1745)^a^
Age of parent (years), mean (SE)		32.24 (6.69)	—
Age of youngest child (years), mean (SE)		2.50 (3.78)	8.31 (0.14)
Number of children, mean (SE)		—	1.84 (0.02)
**Age group of parent (years), n (%)**			
	<30	395 (36.17)	57 (5.07)
	30-34	356 (32.60)	129 (11.97)
	35-39	189 (17.31)	222 (19.66)
	40-44	96 (8.79)	244 (22.61)
	≥45	56 (5.13)	430 (40.69)
	Missing, n	5	663
**Number of children, n (%)**			
	1	492 (44.85)	—
	2	402 (36.65)	—
	3	147 (13.40)	—
	4	44 (4.01)	—
	5	5 (0.46)	—
	≥6	7 (0.64)	—
**Sex of parent, n (%)**			
	Male	80 (7.39)	711 (41.02)
	Female	1003 (92.61)	1034 (58.98)
	Missing, n	14	—
Education level, n (%)			
	High school or less	172 (16.09)	358 (20.41)
	Trade or vocational	286 (26.75)	514 (29.63)
	Some university	110 (10.29)	144 (7.75)
	Bachelor’s or graduate degree or professional certification	501 (46.87)	722 (42.19)
	Missing, n	28	7
**Household income level (Can$), n (%)**			
	<$30,000	85 (8.6)	157 (9.89)
	$30,000-$59,999	170 (17.3)	385 (24.52)
	$60,000-$99,999	351 (35.6)	498 (32.09)
	≥$100,000	379 (38.5)	519 (33.50)
	Missing, n	112	186
**Region of residence, n (%)**			
	British Colombia	160 (14.61)	175 (10.41)
	Alberta	259 (23.65)	200 (10.17)
	Saskatchewan and Manitoba	137 (12.51)	197 (6.50)
	Ontario	336 (30.68)	486 (38.19)
	Québec	120 (10.96)	427 (24.26)
	Atlantic/Territories	83 (7.58)	260 (10.47)
**Use of Internet to search for information on vaccines (exposure), n (%)**			
	Used the Internet	427 (39.10)	716 (41.57)
	Did not use the Internet	665 (60.90)	1013 (58.43)
	Missing, n	5	16
**Perception on safety of childhood immunizations (outcome), n (%)**			
	1 (Not at all safe)	49 (4.49)	43 (2.49)
	2	48 (4.40)	24 (1.39)
	3	64 (5.87)	50 (2.89)
	4 (Moderately safe)	131 (12.01)	207 (11.97)
	5	134 (12.28)	275 (15.90)
	6	338 (30.98)	500 (28.92)
	7 (Extremely safe)	327 (29.97)	630 (36.44)
	Missing, n	6	16
**Perceived reliability of Internet relative to family/ friends/health care/other, n (%)**			
	Most reliable	64 (5.88)	149 (8.80)
	Second most reliable	97 (8.91)	282 (16.82)
	Third most reliable	123 (11.29)	30 (1.78)
	Not in top three choices	805 (73.92)	1247 (72.60)
	Missing, n	8	37

^a^RDD: random digit dialing. Percentages for the population-based RDD survey are weighted.

**Table 2:** Adjusted cumulative odds ratios of proportional odds logistic regression analysis for the association between parental Internet use to search for information on immunizations and parental perception on safety of childhood immunizations.

Variables			Online survey (n=966) OR (95% CI)	Population-based RDD^a^ survey (n=951) OR (95% CI)
**Predictor of interest**				
	Use of the Internet		1.61 (1.25-2.09)	1.99 (1.55-2.54)
	Did not use the Internet		1.00 Reference	1.00 Reference
**Confounders**				
	**Income level (Can$)**			
		<$30,000	1.42 (0.91-2.21)	1.60 (1.03-2.48)
		$30,000 to $59,999	1.67 (1.20-2.33)	1.19 (0.86-1.63)
		$60,000 to $99,999	1.23 (0.94-1.62)	1.10 (0.82-1.47)
		≥$100,000	1.00 Reference	1.00 Reference
	**Perceived Internet reliability**			
		Most reliable	4.77 (2.88-7.91)	2.18 (1.41-3.36)
		Second most reliable	3.96 (2.58-6.07)	1.12 (0.81-1.57)
		Third most reliable	1.12 (0.78-1.62)	1.66 (0.61-4.50)
		Not in top three choices	1.00 Reference	1.00 Reference	
	Age of parent (continuous)		0.98 (0.96-0.99)	—
	**Age of parent (categorical)**			
		<30	—	1.71 (0.98-2.98)
		30-34	—	0.99 (0.68-1.45)
		35-39	—	1.20 (0.87-1.67)
		40-44	—	1.16 (0.86-1.57)
		≥45	—	1.0 Reference
	**Region of residence**			
		British Colombia	0.93 (0.65-1.33)	1.63 (1.04-2.57)
		Alberta	0.77 (0.56-1.06)	1.38 (0.89-2.15)
		Saskatchewan and Manitoba	0.64 (0.43-0.95)	1.71 (1.13-2.59)
		Ontario	1.00 Reference	1.00 Reference
		Québec	1.89 (1.27-2.83)	1.26 (0.89-1.78)
		Atlantic/Territories	1.00 (0.63-1.60)	1.08 (0.75-1.56)

^a^RDD: random digit dialing.

## Discussion

Although the Internet has been reported as an important influence on parental perception of risk on childhood immunizations, to our knowledge no study has quantified the association between seeking vaccine information on the Internet and perception on safety of childhood immunizations among Canadian parents. The analyses on both datasets resulted in the same conclusion with similar effect sizes not significantly different from one another. The findings from both data sources confirm the assumed relationship between looking for vaccine information on the Internet and perception of risk on vaccine safety, with both samples revealing higher odds of perceiving vaccines as “not safe” in parents who used the Internet to search for information on vaccines compared to parents who did not use the Internet for vaccine information. These results are consistent with a before-and-after Internet experiment study conducted in Germany where participants exposed to short searches on vaccine critical websites reported an increase perceived risk of vaccinating [39].

This study utilized two different data sources on Canadian parents, sampled at different times. The RDD data were collected in March 2011 and the Web-based data were collected between December 2013 and January 2014, thus the results represent a specific period in time. To our knowledge, there have been no significant policy changes from 2011 to 2014 and although several measles outbreaks have occurred since early 2011, both populations would have been exposed to the media coverage. Respondents were also asked about factors influencing vaccination decisions and there was no significant difference in time-related contextual influences reported. Furthermore, we received similar results in both samples, thus the bias introduced by time-varying contextual influences is likely nondifferential.

Due to incomplete and unreliable data, our study could not account for the reliability of the websites parents searched or in the type of communications they were exposed to on the Internet. For example, many Web-based respondents reported using search engines and clicking on the websites from their search results, as opposed to identifying specific websites or types of websites. According to the summary report by the research company who conducted the RDD survey, “Google search engine” was the primary website reported to be used by almost half of the respondents who searched for vaccination information online, followed by various government websites and other websites such as medical sites (eg, WebMD), online chat rooms, wikis, etc [29]. Thus our respondents were likely exposed to a variety of messages, but several studies have shown an abundance of antivaccination messaging via Internet searching (or “Googling”) for information on vaccines [40-43]. In addition, this study did not take into account the respondents’ perceptions of risk on vaccine safety prior to the Internet search, and if the Internet altered prior perceptions of risk or acted to support previously held beliefs. Thus, we can establish a significant association between parents seeking vaccine information online and negative perception of risk on childhood immunizations; however, we cannot establish causality or direction of causality.

As more people abandon landline telephones, the validity of traditional population telephone surveys is compromised with low response rates and potentially nonrepresentative samples. Representativeness and validity concerns are also relevant for Web-based surveys as research relies on the collection of self-reported data by self-selected online participants [44]. Both sampling techniques produced low response rates of 23%, which could produce biased samples; however, analysis of the two different samples via two different regression methods produced similar models and conclusions indicating the results were likely not due to chance. In addition, the Web-based sample achieved a similar or better response rate to other studies using Web-based recruitment [27], and the increase in cell phone utilization and call display presents a challenge in preventing noncoverage bias in the RDD sample [45]. Furthermore, the intent of the study was not to generalize the results to Canadian parents but to have sufficient power to examine the relationship between the predictor and the outcome. Thus, the results from our primary data collection can only be applied to our sampled Web-based population and not generalizable to the Canadian population. Nonetheless, we had similar results in the Web-based sample as the RDD sample, which was intended to be representative of Canadian parents.

Current initiatives aiming to reach and influence parents’ decision to vaccinate have not adequately abated the influence of the online antivaccination movement. Health agencies currently have an online presence; however, they have been slow to fully adopt the true nature of social media platforms and communication remains mostly by top-down dissemination of information [18,46]. However, studies have shown that health communications in the form of stories or testimonials are important influences on risk perception [39,47] and that there is a need for more dialogue-based approaches targeted to specific subpopulations [48]. As evidenced in this study, using the Internet for vaccination information and the relative importance of the Internet as a trustworthy and reliable source are important factors in individual perception of vaccine safety. The evidence provided here suggests the need for increased efforts in Web-based interventions that promote confidence in immunization. In Canada, search terms of “vaccine,” “vaccination,” and “immunization” via Google will produce more provaccination than antivaccination websites [19] and lead to highly placed sites with significant authority. However, these sites do not meet user expectation of more complex interaction tools and engagement. In addition, mistrust in health care professionals and the government has been reported as an important factor in vaccine hesitancy or refusal [49-51], thus trusted authorities could consider working with other popular websites and influential platforms (such as “Mommy blogs”) to provide information supportive of immunization. Health authorities need to tackle the negative influence of online vaccine information or communications, and better utilize social media for positive communication to reach and influence vaccine-hesitant Canadian parents searching for information on the Internet. The Internet has become an important risk factor for vaccine hesitancy, with exposure nearly doubling the risk that parents will question the value of immunization. This study provides evidence that searching the Internet for vaccination information is significantly associated with Canadian parents’ negative perception of risk on childhood immunizations, thus there is a need for improved Web-based interventions by public health professionals to better understand and mitigate this risk.

## Abbreviations

MMR: measles-mumps-rubella
PHAC: Public Health Agency of Canada
RDD: random digit dialing
